# Editorial: The role of artificial intelligence technologies in revolutionizing and aiding cardiovascular medicine

**DOI:** 10.3389/fcvm.2025.1588983

**Published:** 2025-04-04

**Authors:** Omneya Attallah, Xianghong Ma, Mohamed Sedky

**Affiliations:** ^1^ Department of Electronics and Communications Engineering, College of Engineering and Technology, Arab Academy for Science, Technology and Maritime Transport, Alexandria, Egypt; ^2^ Wearables, Biosensing, and Biosignal Processing Laboratory, Arab Academy for Science, Technology and Maritime Transport, Alexandria, Egypt; ^3^ College of Engineering and Physical Sciences, Aston University, Birmingham, United Kingdom; ^4^ Faculty of Computing, Engineering and Sciences, Staffordshire University, Stoke-on-Trent, United Kingdom

**Keywords:** artificial intelligence, cardiovasccular disease, cardiovasccular medicine, machine learning (ML), deep learning, language models (LMs), natural language processing, federated learning (FedML)

**Editorial on the Research Topic**
The role of artificial intelligence technologies in revolutionizing and aiding cardiovascular medicine

## 1 Introduction

Cardiovascular disease (CVD) continues to be the foremost worldwide contributor to mortality and morbidity (1), accounting for roughly 17.9 million fatalities each year—a burden anticipated to increase due to aging populations and lifestyle modifications (2). Despite progress in prevention, diagnosis, and treatment, the application of research in clinical practice continues to be difficult, as cardiologists face complicated decision-making amidst varied patient requirements, swiftly changing evidence, and the incorporation of multimodal data from high-resolution imaging and ongoing biomedical signal tracking (3). The exponential increase in medical data and the advancement of diagnostic tools present a potential for enhancing care, yet they also pose challenges in providing timely and personalised interventions. Artificial intelligence (AI) has been recognised as a transformative technology capable of improving diagnostic precision, optimising treatment methodologies, and ultimately alleviating the worldwide incidence of CVD by connecting research innovation with practical clinical application (4).

AI encompasses a variety of technologies that have rapidly progressed to improve individual decision-making and address important issues in cardiovascular care, including machine learning, deep learning, computer vision, pattern recognition, federated learning, natural language processing, and generative AI (5). Traditionally, the diagnosis and treatment of cardiovascular diseases have mostly relied on conventional approaches, which usually face restrictions in accuracy, fast identification, and tailored strategies for treatment (6). The utilisation of AI in cardiology takes note of an unprecedented evolution in the manner of data-driven decisions and predictive models leading to personalised medicine tailored to the individual preferences of each patient (7). Utilising extensive data obtained from various sources, including electronic health records (EHRs), medical imaging (MRI, CT), genomic profiles, wearable technology, biosignals (ECG, PPG), and real-time tracking platforms (8), AI makes limitless breakthroughs in deciphering complex patterns concerning population demographics (6) by means of computational power to evaluate these massive datasets. For example, AI-predictive models can predict the chances of clinical-anatomical intervention after endovascular repair (9, 10). Additionally, computer-aided diagnostic systems based on deep learning techniques assist in diagnosing myocardial infarction (MI) in its early stages using MRI scans (11). Moreover, ML-based analytics, in conjunction with biosignals such as the ECG, can detect cardiac abnormalities in fetal during pregnancy (12). This change in technology is particularly of utmost importance with the rapid growth of wearable AI-based devices like smartwatches that support atrial fibrillation (AF) detection and treatment in real-time (13). These advances help to enhance both risk stratification and diagnosis accuracy for personalised outcome predictions such as recurrent heart failure or post-interventional effects (3).

Advanced AI technologies are also going to enhance research and clinical programs within cardiology to achieve validity, efficiency, and improved patient-centered care AI drives a transformative impact on cardiovascular healthcare including drug discovery and development, risk profiling, predictive analysis, and clinical decision support system advancement. However, reaching the full potential of AI in cardiology requires synergistic cooperation among cardiologists, computer scientists, and biomedical engineers to create comprehensive models with multiple data types while upholding ethical standards, accountability, and repeatability (14). The conjoining of precision medicine, the Internet of Medical Things (IoMT), and AI holds remarkable potential to change the way healthcare is delivered (15). Harnessing such synergies leads us to a future where health management can enhance clinical decision-making.

This editorial highlights the state-of-the-art applications of AI in cardiovascular medicine with a focus on AI technologies implemented in studies published in the section of the Research Topic in the Frontiers in Cardiovascular Medicine Journal. From early diagnosis to outcome prediction, this editorial aims at the potential uses of these approaches in solving major cardiologic challenges and discusses the possible effects of their application on patients and physicians. There is an appeal for further investigations and innovation in the evolving discipline.

## 2 An overview of the studies that contributed to this research topic

On this research topic, twenty articles were received. Each article submitted to the Research topic went through an exhaustive review procedure including at least two reviewers and two rounds of extensive edits prior to acceptance. Nine papers in all—two systematic reviews, one study protocol, and six original research publications—were chosen. The following is a list of the works significantly advancing this area of study.

The AI-based identification of atrial fibrillation during sinus rhythm (AIAFib) trial is a prime example of how AI might transform AF management by filling in a major diagnostic void. Early identification is difficult even with AF's great morbidity and death risks, especially in sinus rhythm (SR) when paroxysmal AF (PAF) prevents traditional techniques for diagnosis. Targeting to predict PAF during SR, the multicenter retrospective study protocol (Baek et al.), uses a deep learning method to analyse >50,000 12-lead ECGs from 10 Korean tertiary hospitals, so linking clinical findings such hospitalisation, procedural interventions (e.g., ablation), and mortality with risk scores determined by AI. The trial aims to verify not only the prognostic value in real-world cohorts but also confirm the diagnostic accuracy of the algorithm by using strict multivariate Cox regression and survival analyses. If effective, AIAFib might restructure risk stratification paradigms, enable earlier, data-driven interventions, and underline AI's ability to turn ordinary ECGs into potent predictive tools. This protocol fits a larger movement towards AI-enhanced precision cardiology, in which scalable computational methods and multimodal analysis of information promise to maximise findings for cardiovascular diseases worldwide.

The systematic review and meta-analysis (Fadilah et al.) assess the diagnostic efficacy of ML methods integrated with diverse non-invasive modalities for the diagnosis of pulmonary arterial hypertension (PAH), a critical cardiovascular disorder conventionally diagnosed through the invasive gold standard of right heart catheterisation (RHC). The study investigates the accuracy of techniques such as electrocardiogram (ECG), echocardiogram hematological biomarker, and imaging modalities like chest x-ray, CT, and MRI when employed with AI. Analysing 26 studies, the authors used STATA V.12.0 for meta-analysis and the Quality Assessment of Diagnostic Accuracy Studies-2 (QUADAS-2) tool for quality evaluation and found an overall sensitivity of 81%, specificity of 84%, and an area under the curve (AUC) of 89%. While ECG showed similar accuracy, echocardiograpm showed amazing performance with a sensitivity of 83% and a specificity of 93%. With their lowest negative likelihood ratio (NRR), blood biomarkers proved useful in PAH exclusion. These results show that ML-enhanced echocardiogram and ECG could be interesting, less invasive substitutes for RHC, so improving accessibility and safety in PAH diagnosis and hence preserving high diagnostic accuracy (AUC of 89%).

In addition, the systematic review (Chavez-Ecos et al.) provides comprehensive coverage of the quality and efficacy of Mobile Health Applications (MHAs) intended for cardiovascular risk assessment by healthcare practitioners. The methodology used in this research included applications available on major digital markets (Apple Store, Play Store, and Microsoft Store) as relevant as of August 2023. These were analyzed against recognised clinical protocols from the European Society of Cardiology and various risk approaches including the Framingham Risk Score and Atherosclerotic Cardiovascular Disease (ASCVD) score. The researchers applied the validated Mobile Apps Rating Scale (MARS) and the IMS Institute for Healthcare Informatics functionality scale and they discovered that only six of the 18 applications reached the highest rankings in quality with ESC CVD Risk Estimation and ASCVD Risk Estimator Plus being considered “the best”. In spite of the growing demand for mHealth tools, it points out the major gap in the supply of quality, evidence-based applications for healthcare professionals dealing with cardiovascular risk evaluation; the call for improvement in app design is for incorporation of collaborative decision-making modalities for the clinicians. On the one hand, the research promises big, while on the other what comes through is the challenge in assuring that these technologies work reliably and are usable in real-world contexts.

The study (Yordanov et al.), on the other hand, concerns the empirical assessment of the efficacy of federated learning (FL) algorithms in developing clinical forecasting algorithms related to the thirty-day death risk of individuals experiencing transcatheter aortic valve implantation (TAVI). The research studied through four multicentric modeling perspectives employing data spanning sixteen Dutch TAVI hospitals from 2013 to 2021-entail ensemble: aggregating predictions from local models; federated averaging (FedAvg): gathering model predictions without distributing raw data; local: separate models per hospital; and central: a single model learned on combined data. These results show that during internal validation, FedAvg and ensemble outperformed the local strategy while matching the predictive performance of the central strategy (AUC = 0.67–0.68). Interestingly, both FedAvg and ensemble performed similarly to the central against external geographic validation with slight calibration variations. This finding indicates that FL methods retain generalisability and predictive accuracy without reliance on pooling data, and adequately handling privacy issues while permitting joint model development among institutions. This is a pivotal work demonstrating the feasibility of FL as a viable alternative to centralized training procedures in clinical settings, opening the way to further common acceptance in cardiovascular research and beyond.

Another article on this research topic (Shiferaw et al.) provides an extensive evaluation of the rapid-moving contribution of AI to CVD research. Using topic modeling based on latent Dirichlet allocation (LDA) and bibliometric analysis of 23,846 studies from the Web of Science and PubMed, the authors of this article map major issues, trends, and collaborations influencing this ever-evolving field. Driven mostly by contributions from nations including the USA, China, India, the UK, and Korea, the investigation notes an exponential rise in AI-related CVD research. The UK, Germany, and Australia especially lead in worldwide cooperative projects. Along with developing areas like “robotic-assisted cardiac surgery,” “stroke and robotic rehabilitation therapy,” “cardiac image analysis,”, and “retinal image analysis and CVD,” significant areas of study recognised are “biomarker and wearable signal analyses.” Emerging as major methods are convolutional neural networks, long short-term memory (LSTM), and K-nearest neighbor (KNN), which reflect growing attention on neural networks and cutting-edge imaging methods. This paper not only emphasises the transforming possibilities of AI in improving diagnosis, treatment, and patient outcomes but additionally provides a useful tool for researchers, doctors, and policymakers trying to negotiate and maximise the future paths of artificial intelligence in preventing CVD.

The work (Amadou et al.) addresses a long-standing obstacle in cardiac imaging: operator-dependent variations in ultrasonic acquisition, which usually reduces diagnostic consistency. This research uses a GPU-accelerated simulation process that synthesises patient-specific, view-dependent ultrasonic images from multi-modal segmentations (e.g., MRI/CT), so enabling adaptable training of AI models for autonomous probe navigating tasks. The synthetic dataset, which was created from over 1,000 patient anatomy, was validated using phantom experimentation and real-world echocardiography view classification tasks. It showed its ability to improve neural network efficiency, especially for insufficiently represented perspectives where real-world data is limited. This significant development not only avoids the “data hunger” of conventional AI algorithms but also democratises the availability of high-quality imaging knowledge, lowering dependency on operator ability. The research advances AI's role in standardising cardiac ultrasonic by addressing simulation-to-reality gaps, so opening the path for autonomous systems that might transform point-of-care diagnostics and interventional recommendations in resource-limited environments.

This study (Kirdeev et al.) examines significant deficiencies in personalised medicine by investigating the incorporation of genetic factors into prognostic models for MI individuals. The research employs ML methods to forecast major adverse cardiac events (MACEs) employing clinical, imaging, laboratory, and genetic data from 218 MI patients over a 9-year follow-up duration. The findings illustrate the importance of the VEGFR-2 genotype (rs2305948) as one of the five best-predictive attributes, in conjunction with statin dosage, coronary artery lesions, left ventricular parameters, diabetes status, type of revascularisation, and age. The CatBoost algorithm, enhanced through sequential feature selection (SFS), attained a remarkable AUC of 0.813 on the test cohort, demonstrating the model's efficacy. Applying the SHapley Additive exPlanationsis (SHAP), a model-agnostic method elucidates additive risk contributions, facilitating the precise stratification of high-risk subgroups, underscoring how ML can customise post-MI care. This study illustrates how AI can integrate omni-omics data with biological, biochemical, and imaging, advancing personalised risk prediction by linking the insights of the molecular to therapeutic decision-making-prime innovations that could optimise additional preventive efforts in cardiology. This new strategy highlights the role of genetic factors in assessing cardiovascular risk while accentuating the true potential of AI to advance precision medicine in patients suffering from myocardial infarction.

In addition, (Tang et al.) provide a comprehensive overview of the use of ML and data mining techniques to evaluate the risk of in-hospital death in patients with acute ST-elevation MI (STEMI) following primary percutaneous coronary intervention (PCI). The work investigates several gradient-boosting methods. Furthermore, feature selection methods including SHAP and recursive feature elimination (RFE) are applied. The study uses a large database including 4,677 people from a regional vascular centre in Vladivostok between 2015 and 2021. To improve and increase the predictive power, hybrid algorithms also combine metaheuristic optimisation. With the extreme gradient boosting, optimised with bald eagle search optimiser, these hybrid techniques are shown to be able to stretch beyond more than the conventional global registry of acute coronary events (GRACE) score and produce recall up to 0.99. This study would be a suitable tool for informed decision-making for patient treatment and enhanced outcomes in the cardiovascular sector since it shows the application of ML techniques to increase a clinician's capacity to derive better assessments of mortality risk and, hence, would be an appropriate tool.

The final study on this research topic is (Seki et al.), which explores zero-shot visual question answering (VQA's) adoption with multimodal large language models (LLMs) for interpreting 12-lead ECG images. The research showcases the potential of VQA in clinical diagnosis while earmarking crucial drawbacks. Although multimodal LLMs are proficient in logical reasoning and conveying certain assumptions therein, recurrent instances of failing to properly elicit and express these image features from ECGs were accepted as contributing keys to answer selection and producing incorrect answers upon the availability of correct final options. Findings suggest that the model-induced “image hallucinations” are the reason behind the incorrect deductions about visual data and that traditional performance metrics, like percentage correct on multiple-choice items, may poorly reflect a certain architecture of performance complexity with VQA in fine clinics. It does show the established advantages and perceivable disadvantages of multimodal LLMs in medical imaging analysis so that future questions can enhance the pace and contribute to precision and reliability in practical applications.

## 3 Conclusion

The integration of AI in cardiovascular medicine may serve as a novel transformation in disease prevention, diagnosis, and treatment, as exhibited by studies conducted on this Research Topic. These articles collectively highlight AI's revolutionary capabilities of improving early detection of AF by means of AI-augmented ECGs, better non-invasive diagnosis of pulmonary arterial hypertension, optimisation of risk stratification for important adverse cardiovascular events post-MI, and prediction of in-hospital mortality following percutaneous coronary intervention. Innovations, such as FL and synthetic ultrasound simulation, demonstrate the ability of AI to tackle data privacy and scarcity issues without compromising clinical accuracy. Simple as this may appear, these studies remain constrained by the significant limitations of AI-based systems. Many models are only able to leverage multiple algorithms within data silos. Algorithmic biases and inadequate external validation limit traditional models, while multimodal large language models reveal dangers of critical interpretation “hallucination”.

Furthermore, there stand major barriers to the sustainability of integration of AI into standard practices, namely harmonising data disparities leveraging privacy-preserving systems, departing from accuracy scores to standard assessment metrics, and achieving interpretability for bolstered clinician trust. In addition, the disparity between technocentric agility and applicable clinical processes, aptly illustrated through shortcomings in the mHealth application capabilities, demands better cooperation from engineers, practitioners of healthcare, and policymakers.

Further efforts should emphasise multimodal AI algorithms that incorporate genomics, imaging, wearables, and EHR into comprehensive predictive frameworks. Progress in compact structures (e.g., MobileViT, EfficientFormer) and synthesised data generation may facilitate broader access to AI tools, especially in resource-constrained environments. Concurrently, ethical frameworks for bias reduction, regulatory supervision, and patient-centered design are essential for ensuring equitable adoption. The bibliometric examination indicates that the rapid expansion of AI-CVD research requires international collaboration to standardise practices and expedite translation. By overcoming these challenges, AI can advance beyond its current function as a decision-support instrument, becoming a fundamental element of precision cardiology—one that enables clinicians to customise care and ultimately transform cardiovascular outcomes globally.

## References

[1] Roth GA Mensah GA Johnson CO Addolorato G Ammirati E Baddour LM Global burden of cardiovascular diseases and risk factors, 1990–2019. J Am Coll Cardiol. (2020) 76:2982–3021. 10.1016/j.jacc.2020.11.010 33309175 PMC7755038

[2] World Health Organization. Cardiovascular diseases (CVDs) (2021). Available at: https://www.who.int/news-room/fact-sheets/detail/cardiovascular-diseases-(cvds) (Accessed February 20, 2025).

[3] Johnson KW Torres Soto J Glicksberg BS Shameer K Miotto R Ali M Artificial intelligence in cardiology. J Am Coll Cardiol. (2018) 71:2668–79. 10.1016/j.jacc.2018.03.521 29880128

[4] Lopez-Jimenez F Attia Z Arruda-Olson AM Carter R Chareonthaitawee P Jouni H Artificial intelligence in cardiology: present and future. Mayo Clin Proc. (2020) 95(5):1015–39. 10.1016/j.mayocp.2020.01.038 32370835

[5] Haq IU Chhatwal K Sanaka K Xu B. Artificial intelligence in cardiovascular medicine: current insights and future prospects. Vasc Health Risk Manag. (2022) 18:517–28. 10.2147/VHRM.S279337 35855754 PMC9288176

[6] Olawade DB Aderinto N Olatunji G Kokori E David-Olawade AC Hadi M. Advancements and applications of artificial intelligence in cardiology: current trends and future prospects. J Med Surg Public Health. (2024) 3:100109. 10.1016/j.glmedi.2024.100109

[7] Olawade DB Wada OJ David-Olawade AC Kunonga E Abaire O Ling J. Using artificial intelligence to improve public health: a narrative review. Front Public Health. (2023) 11:1196397. 10.3389/fpubh.2023.1196397 37954052 PMC10637620

[8] Gupta MD Kunal S Girish MP Gupta A Yadav R. Artificial intelligence in cardiology: the past, present and future. Indian Heart J*.* (2022) 74:265–9. Available at: https://www.sciencedirect.com/science/article/pii/S0019483222001079 (Accessed February 22, 2025). 10.1016/j.ihj.2022.07.004 35917970 PMC9453051

[9] Attallah O Ma X. Bayesian neural network approach for determining the risk of re-intervention after endovascular aortic aneurysm repair. Proc Inst Mech Eng H. (2014) 228:857–66. 10.1177/0954411914549980 25212212

[10] Karthikesalingam A Attallah O Ma X Bahia SS Thompson L Vidal-Diez A An artificial neural network stratifies the risks of reintervention and mortality after endovascular aneurysm repair; a retrospective observational study. PLoS One. (2015) 10:e0129024. 10.1371/journal.pone.0129024 26176943 PMC4503678

[11] Attallah O Ragab DA. Auto-MyIn: automatic diagnosis of myocardial infarction via multiple GLCMs, CNNs, and SVMs. Biomed Signal Process Control. (2023) 80:104273. 10.1016/j.bspc.2022.104273

[12] Al-Saadany D Attallah O Elzaafarany K Nasser AAA. A machine learning framework for fetal arrhythmia detection via single ECG electrode. In: Groen D De Mulatier C Paszynski M Krzhizhanovskaya VV Dongarra JJ Sloot PMA, editors. Computational Science – ICCS 2022. Cham: Springer International Publishing (2022). p. 546–53. 10.1007/978-3-031-08754-7_60

[13] Tison GH Sanchez JM Ballinger B Singh A Olgin JE Pletcher MJ Passive detection of atrial fibrillation using a commercially available smartwatch. JAMA Cardiol. (2018) 3:409–16. 10.1001/jamacardio.2018.0136 29562087 PMC5875390

[14] Gerke S Minssen T Cohen G. Ethical and legal challenges of artificial intelligence-driven healthcare. In: Bohr A, Memarzadeh K, editors. Artificial Intelligence in Healthcare. London: Elsevier (2020). p. 295–336. Available at: https://www.sciencedirect.com/science/article/pii/B9780128184387000125 (Accessed February 22, 2025).

[15] Topol EJ. High-performance medicine: the convergence of human and artificial intelligence. Nat Med. (2019) 25:44–56. 10.1038/s41591-018-0300-7 30617339

